# Co-creation of health-enabling initiatives in food retail: academic perspectives

**DOI:** 10.1186/s12889-023-15771-z

**Published:** 2023-05-25

**Authors:** Carmen Vargas, Julie Brimblecombe, Steven Allender, Jillian Whelan

**Affiliations:** 1 Global Centre for Preventive Health and Nutrition (GLOBE), Institute for Health Transformation, School of Health and Social Development, Deakin University, Geelong, VIC Australia; 2 Department of Nutrition, Dietetics and Food, School of Clinical Sciences, Monash University Clayton, Clayton, VIC Australia; 3 Locked Bag 20000, Geelong, VIC 3220 Australia

**Keywords:** Co-creation, Food retail, Healthy food environment, Qualitative research

## Abstract

**Introduction:**

Co-creation of healthy food retail comprises the systematic collaboration between retailers, academics and other stakeholders to improve the healthiness of food retail environments. Research into the co-creation of healthy food retail is in its early stages. Knowledge of the roles and motivations of stakeholders in intervention design, implementation and evaluation can inform successful co-creation initiatives. This study presents academic experiences of stakeholder roles and motivations in the co-creation of healthy food retail environments.

**Methods:**

Purposive sampling of academics with research experience in the co-creation of healthy food retail initiatives. Semi-structured interviews conducted between October and December 2021 gathered participants’ experiences of multi-stakeholder collaborative research. Thematic analysis identified enablers, barriers, motivations, lessons and considerations for future co-creation of healthy food retail.

**Results:**

Nine interviewees provided diverse views and applications of co-creation research in food retail environments. Ten themes were grouped into three overarching areas: (i) identification of stakeholders required for changes to healthier food retail; (ii) motivations and interactions, which included the intrinsic desire to build healthier communities along with recognition of their work; and (iii) barriers and enablers included adequate resourcing, effective and trusting working relationships and open communications.

**Conclusion:**

This study provides insights that could help future co-creation in healthy food retail environments. Trusting and respectful relationships and reciprocal acknowledgement between stakeholders are key practices in the co-creation process. These constructs should be considered in developing and testing a model that helps to systematically co-create healthy food retail initiatives that ensure all parties meet their needs while also delivering research outcomes.

**Supplementary Information:**

The online version contains supplementary material available at 10.1186/s12889-023-15771-z.

## Background

Unhealthy diets are associated with chronic disease morbidity and mortality worldwide [1]. Actively addressing food environments [2, 3] to create opportunities to achieve healthy, accessible and affordable diets represents a critical field in population health [4–7]. Reports have shown that dietary quality can be shaped by an individual’s access to food sources within food environments and the in-store characteristics that affect food selection [8–10]. As a result, food retail outlets (e.g., supermarkets, grocery and food stores) may influence health behaviours by enabling or limiting the presence of “healthy” or “unhealthy” food offerings within food outlets [11–13]; making them strategic settings for health-enabling initiatives [8, 9, 14–17].

Health-enabling initiatives in food retail outlets have attempted to enhance customers’ dietary behaviour at the point of sale through complex initiatives [7, 18, 19]. Yet there is no consensus on the most relevant type of strategy or variable(s) to consider [4–7] (e.g., price promotion, store design and product placement strategies or health promotion). Additionally, these initiatives that aim to improve dietary behaviour are only sometimes sustainable over the long term [7, 18, 19]. Cross-sector collaboration (e.g., government, the private sector and civil society) is required to maximise the impact of health prevention initiatives [14], including those related to food retail [4, 19, 20].

To date, research has demonstrated the importance of engagement with food retailers (i.e., store owners and managers) [4, 19, 21], and their consumers [4] to enhance success in developing health-enabling strategies in food retail outlets [4, 19]. Middle et al [19]. reported enablers and barriers to implement health-enabling food retail store initiatives (e.g., setting characteristics, costs and benefits, and capacity-building support). They concluded that strategies must be aligned with the interests of all the parties involved (researchers and non-researchers) to overcome barriers to health-enabling initiatives in food retail outlets. Therefore, transdisciplinary collaboration is critical to improving the food retail environment, and co-creation may provide a means to understand and optimise these strategies.

Co-creation is a collaborative approach of creative problem-solving between diverse stakeholders, from problem identification and solution generation to implementation and evaluation stages [22–24]. It has shown positive influences in diverse disciplines (e.g., education [25, 26], planning and development studies [27], community-based research [28, 29], sustainability of health care services [29, 30] and health promotion [22]) for over 50 years, yet It has gained relatively recent recognition [31]. Co-creation is recognised because it is a participatory, collaborative, context-sensitive and knowledge-based practice [22, 29], where actors collaborate with different knowledge, resources and competencies to solve a shared problem [32]. Co-design is a method commonly used in co-creation related to the design of a strategy that positions participants’ needs, expertise and knowledge at its centre [23, 33]. Within the co-creation landscape, individuals involved in the development, implementation and evaluation of initiatives have been described in diverse ways (e.g., end-users, consumers, customers, citizens, or community members) [29, 34–36]. Within this study, we have used the term ‘consumers’ to include customers and community members who purchase products in these food retail outlets.

For the food retail setting, co-creation provides a way to systematically understand the collaboration between diverse stakeholders to improve the healthiness of food retail outlets. Some studies reported parallel benefits of collaboration between diverse stakeholders (i.e., suppliers, retailers, community, government) with co-created and tailored interventions that target specific participants and settings [37, 38]. In the food retail setting, the multi-stakeholder collaboration represented that non-researchers provided contextual knowledge of this emerging area of public health. Researchers’ skills strengthened the successful design, implementation and evaluation of health-enabling initiatives [39]. Thus, co-creation enables retailers, researchers, consumers and other interested parties to construct a shared goal that facilitates the design and implementation of health-enabling food retail initiatives [19]. When done well, the co-creation approach meets the needs of all involved groups [19, 37].

A recent systematic review identified that co-creation is mostly used when developing health-enabling food retail initiatives with particular population groups, such as lower-socio-economic and Indigenous communities, where the needs and perspectives of these often-marginalised groups may be overlooked. Key elements of co-creation were identified, such as the type of stakeholders involved, their level of engagement and their motivations [39]. Despite the increased application of co-creation in health more broadly, there needs to be more peer-reviewed literature on utilising co-creation concepts to achieve mutually beneficial outcomes in food retail outlets [19, 30].

The lack of research regarding ways to systematically use co-creation as an approach in food retail means academics are currently the main group investigating how to strengthen the successful co-creation of health-enabling initiatives in this setting [40]. For this reason, researcher perceptions are beneficial to advance co-creation research. This paper reports on a study undertaken to elicit academics’ perceptions on the type and roles of the stakeholders involved in a co-creation process related to a healthy food retail initiative, motivations to be involved in health-enabling initiatives, and barriers and enablers concerning the implementation of these initiatives. This study aimed to understand the experiences, roles, and motivations for the co-creation of health-enabling initiatives from stakeholders beyond retailers. In this paper, we address the following research questions from the perspectives of the academics who participated in this study:

- Who should be involved in the co-creation of healthy food retail initiatives?
- How do academics reflect on their role in the process of co-creation of healthy food retail initiatives?
- What factors could motivate a food retailer’s participation in the co-creation of healthy food retail initiatives?
- What are the learnings, enablers and barriers identified to co-create healthy food retail initiatives?

## Methods

### Study design

A qualitative approach was used to obtain detailed insights from academic informants [41]. Semi-structured one-on-one, online interviews were chosen as an acceptable and appropriate method to inform ways to co-create future health-enabling initiatives in food retail outlets [42, 43]. Ethics approval was obtained from the Deakin University Faculty of Health (HEAG-H 63_2021). All participants provided informed consent to be recorded and to use direct quotes in a non-identifiable form. The COnsolidated criteria informed reporting of the study design, results, and analysis for REporting Qualitative research checklist (COREQ) [44] (Additional file Table S1).

### Researcher team and reflexivity

Three of the research team are members of the Institute for Health Transformation at Deakin University, recognised for strong community partnerships and co-designing initiatives with the community. All are members of the Centre of Research Excellence in Food Retail Environments for Health (RE-FRESH APP1152968). The first author (female, a PhD candidate with training and experience in qualitative methods and practice experience as a public health nutritionist) conducted all interviews. The second author is a postdoctoral research fellow (female) conducting research in systems thinking and healthy food environments using implementation science and qualitative methods. The other two members of the research team (one Professor [male] and one Associate Professor [female]) have experience conducting qualitative studies and leading focused research on the prevention of chronic conditions, including addressing determinants of chronic disease, including complex food environments.

### Theoretical Framework

The study adopted a social constructionism paradigm, highlighting the importance of multiple perspectives of reality to generate knowledge [45]. The research team applied this paradigm by examining how participants create meaning of co-creation as an approach to develop health-enabling strategies in food retail outlets through their perspectives and experiences. The organisation and data analysis were interpreted through our understanding and construction of co-creation, which mainly comes from service management and marketing fields and has been adapted to public health initiatives.

### Participants and recruitment

We used purposive sampling to identify and recruit a sample of ‘academic expert informants’ who had previously conducted and published collaborative research on healthy food retail initiatives [43]. A recently published systematic review was used to identify potential participants [39]. From the 23 papers, one study was conducted by a single author who was deceased (no further action was taken), and three authors were repeated as corresponding authors two times. The eligible 19 corresponding authors were contacted via publicly available email addresses and invited to participate in a one-on-one interview via email correspondence detailing the research aims and objectives. From these 19 invitations, three participants declined the invitation but identified three potential participants who were also authors of the same study and provided emails for these additional contacts (n = 19). After researchers accepted to participate, an online meeting (using Zoom App) was set up at an agreed time. The study’s plain language statement and consent form were provided with the meeting invitation.

### Data collection

The primary author conducted one-on-one semi-structured interviews using an interview guide based on the co-creation literature [23, 46]. The interview guide was developed through an iterative approach that included trial and discussion with the research team (CV, JW, JB, and SA), followed by pilot testing and further refinement of the interview guide (Additional file Table S2). Two research team members (CV and JW) pre-tested the final interview schedule. All interviews were conducted in English and audio recorded via Zoom [47] at an agreed time between October and December 2021. During interviews, written notes were taken by the interviewer to capture topics requiring further discussion with subsequent participants. All interviews were transcribed, de-identified, and cross-checked against the recordings. A copy of the interview transcript was returned to each participant for the edit and checking if desired [48]. Two participants reviewed and returned their transcripts with desired amendments and additions. One participant returned the transcript with major edits, which were made and returned to the participant for cross-checking.

### Data analysis

A deductive thematic analysis [41] was used to analyse interview transcripts with the assistance of Nvivo Software [49] for coding and theme development. A codebook was developed to ensure consistent coding between authors and data extraction [50, 51]. A codebook was developed and revised through an iterative process, including regular meetings between the coding team to discuss discrepancies in interpretation until a consensus was reached. An initial list of six codes was created based on the interview guide to start the codebook development. The coding team (CV and JW) tested the codes on a randomly selected interview; codes were discussed and refined accordingly, and new codes were added. The first iteration of the codebook included definitions and differentiation between codes. The coding team (CV and JW) tested the new codes on a second randomly selected interview. After discussing and adjusting the codes, these were clustered to develop themes. Themes were defined, reviewed, and refined to get the final codebook (Additional file Table S3). To determine the reliability of the codebook [51, 52], two researchers (CV and JW) double-coded one transcript independently with a resulting kappa value of 0.75–0.90 on each code. The primary author coded the remaining eight transcripts against the final codebook.

## Results

Of the 23 articles, 19 authors were invited; eight (42%) did not reply to the invitation (including two not operational emails); five (26%) declined due to other commitments, with three of these suggesting an alternate participant that they considered could provide deeper detail of the study or could commit to the interview. In total, nine (47%) participated, including the three suggested academics, in an interview that lasted for 40 to 70 min on average.

Given the specificity of the initiatives, this sample size was considered sufficient to understand health-enabling co-creation research in food retail outlets [53, 54]. The nine participants (women = 7 and men = 2) were researchers within academic institutions using varying methods to co-create health-enabling initiatives in food stores, retail stores, supermarkets, or convenience stores. The settings of these initiatives were diverse, having been conducted among urban areas, Indigenous peoples or low-income communities in western countries (i.e., New Zealand, Australia, the United States of America, and Denmark). The description and analysis of the co-creation of these initiatives have been reported elsewhere [39].

The themes were synthesised narratively and conceptualised into three areas: (i) identification of stakeholders; (ii) motivations and interactions; and (iii) barriers, enablers, and future considerations for implementation. Each theme is described in more detail below.

### Identification of interested stakeholders

Stakeholder identification was a key theme that participants considered important in the co-creation of health-enabling strategies in food stores. Participants identified stakeholders as store owners, consumers, food suppliers, distributors or manufacturers, and researchers. Other stakeholders that might have interest and/or power in healthy food retail initiatives, such as parents, adolescents, schools and health centres, government, retailers or commerce organisations, and community groups, were also identified.

*[…] you would have to really think through with the people that you’re collaborating with, who needs to be sitting at the table? who are the right key stakeholders in order for this to work most effectively? like not just thinking about now, but thinking down the track, thinking about sustainability, thinking about scale-up, who needs to know? who needs to be involved in this collaboration? (Participant 1).*

Groups and networks at a community level were also referred to by participants as providing support to co-created initiatives, whether it was in the engagement, implementation or sustainment stages of a research project. These groups were identified as government agencies, neighbourhood associations, religious institutions, non-government organisations or universities:

*[…] look for any naturally occurring groups of people that would have been working to improve the food environment as one of their areas of interest or focus of their work […] I would first look to see what already exists, what’s already important and try to link in and expand on some of those efforts (Participant 5).*

In determining which stakeholders to engage and why, participants considered each stakeholder’s role and the expected outcomes and objectives of the project, resources (e.g., time, capital) and setting:

*[…] I think the learnings are definitely around what’s doable and who owns that. Then how your agreement will work out, how you work together, I think that’s really important to set up very early in the piece, about how you’re going to work together and who owns what and all that type of thing (Participant 8).*

Participants considered that their role as academics could include underwriting and supporting these initiatives beyond a traditional research relationship, such as overcoming resource implications (e.g., economic, staff). Some participants believed that the definition of roles must have some flexibility, as these can change, evolve or adapt over time:

*I think that defining roles is critical. I think defining them up front sometimes can be a mistake … there needs to be some flexibility because whenever you bring people together, everyone was excited to do it, and the next meeting, they’re not all there, and it’s just finding out to who can be there (Participant 3).*

### Motivations and interactions

Participants reported multiple motivations to co-create healthy food retail outlets related to business and community health outcomes. Participants shared their understanding of food retail outlets as a setting that is also a business that provides income for retailers. To commence the co-creation process to develop health-enabling initiatives in food retail outlets, having a “cause” such as childhood obesity, healthy eating for kids, or environmental sustainability can be helpful. In contrast, smaller independent stores were acknowledged to have often a more intrinsic motivation to support the health of their community.

Other food stores’ motivations to co-create healthy food retail outlets relate to the visibility of the store’s actions, which in turn may improve the store’s image from the community perspective:

*They were very interested in positive media attention, so that was important for them as well, so they really engaged in the health educational activities installed, whereas some of the more placement and promotion they were more like “does it work?” (Participant 9).*

Identifying existing interest groups was a key strategy, particularly in the initiation phase of the co-creation process. Existing strong relationships were essential for effective engagement, as was the identification of existing interest groups with a shared agenda for healthy retail. Participants provided meaningful examples of how to build these trusting relationships that are linked to working with the community, having a process of assigning roles, understanding people’s practical challenges and having different voices represented:

*My experience is that when you have the opportunity to have discussions and conversations with store directors and provide them with the information that can help them make decisions, that seems to make a difference (Participant 1).*

In the prioritisation of strategies to implement in the food retail outlet, participants found that it was crucial to acknowledge the role and needs of retailers. Acknowledging retailers can influence their trust and openness to try new things and will determine the success of an initiative.

*[…] you’re wanting to acknowledge them […] they are trying to make changes […] improve things from their health angle, but you’re wanting to build on it […]. That means that you had to go through that process of establishing relationships again and gaining that consent, building that relationship, building that trust. All of that sort of [things] had to happen before you could move forward (Participant 8).*

Recognising the retailer’s expertise throughout the co-creation process was a tipping point to building trusting relationships. Participants used co-design to find common ground between retailers and health professionals and involve consumers in the co-creation process.

*We put together an idea that we will do co-design sessions with the university and staff of this retailer. We design[ed] a campaign that promotes healthy food choices in the supermarket and then ran a campaign and did an evaluation (Participant 7).*

The most rewarding outcome reported by participants was knowing that capacity has been built through the development of transferable skills. This enhanced capacity shifted their perspectives and attitudes towards a healthy food environment. They experienced more success and better results because they saw value in the co-creation process. This way, there is also a high chance that changes are sustained:

*I think it’s all, its value from an outcome perspective. I think there’s a value from a capacity perspective, I think its value from an engagement perspective, and it goes back to that capacity. I think this is true in most communities; with success, […] is more likely to have success [when] the next problem [comes] (Participant 3).*

### Barriers, enablers, and future considerations for implementation

Participants shared their experiences and perspectives on diverse barriers and enablers related to their initiatives. While many in this group of participants have researched food environments for over twenty years, the main barrier to co-creation was the complexity of the food environment, where continual social challenges occur, and diverse interests are in place. Regarding working with small food store initiatives, the problem lies in scale-up and sustainability.

Participants identified some barriers as “mundane”, such as technological issues (e.g., internet connection, meeting arrangements, supplier issues). These issues, however, were commonly overcome and taken as part of the flexibility that participatory research must have. A common barrier to implementation was the mismatch between the research needs and the retailers’ daily work schedules. Participants described two main difficulties: i) Food retail outlets work at a very fast pace with very limited staff, and any disruption to their routine represents a big effort:

*… in supermarkets, people are extremely busy and can’t just take people out for this; they don’t even usually have a weekly meeting. […] Even the ones who wanted to participate in that said, “we don’t have any time” (Participant 9).*

ii) Many small food stores do not have the systems to measure results (e.g., sales data, stocks) or are not willing to share sensitive information:

*The other interesting thing was that these stores don’t really monitor stock or have like a database, […] it’s all based on looking what you have on the shelf and kind of eyeballing it, which makes data collection (really understanding the business model) really hard (Participant 4).*

Participants recognised three main enablers for effective implementation: (i) resources (i.e., funding, grants) to conduct rigorous studies while reducing the risk for retailers; (ii) effective working relationship with stakeholders that are transparent, trusting and open communication; and (iii) having the right groups of stakeholders involved (e.g., people with skills and knowledge, committed groups).

*I think having an independent party, whether it’s a research team or some other organization it’s helpful to build trust. At least for us between [involved parties] there’s not a common language there, not necessarily common motivations and having someone else that is mediating that, it was really important to them (Participant 4).*

One participant observed that food environment research has evolved from generic and prescriptive initiatives to strategies that involve multiple stakeholders. Collaborating with multiple stakeholders was said to have shown a positive effect, as well as building the capacity of the next generations to continue this work. When prompted, participants acknowledged that policy could play a supportive role in the co-creation of healthier food retail environments. A consistent government policy has the potential to promote equity and support retailers with relevant funding opportunities, yet participants noted that an effective policy requires enforcement.

*if it’s a policy, then it’s applied equally to everybody, and so you don’t have challenges of ‘I stock unhealthy food, and therefore I get more customers. […] The problem with policy seems to be enforcement […] that’s what happened to the [policy example], […] people really didn’t follow it very closely (Participant 5).*

While government policy could regulate food stores, participants suggested support for voluntary organisational policies where food stores can put nutrition policies in place and make alterations within their environments to satisfy their consumers:

*Provide incentives for stores and supermarkets to put a good policy in place so that they can then apply for government funding, approved infrastructure, or more staffing, or that type of thing (Participant 1)*

Participants consider that there is a need to build capacity in the workforce to sustain initiatives through constant coaching and training and to offer feedback and information to store owners in a meaningful and timely way.

*The best advice I can give you is to start with a very small pilot project […]. Do that well, report back to them, show the benefit they got, and that’s how you start building relationships, so they start trusting you (Participant 7).*

Participants recommended that increased application of co-creation was valuable and that collaborative approaches should become the norm. Some participants expressed that working with independent food retail outlets may be the best way to move forward and see changes, as these independent retailers may have more flexibility in their processes and commitment to the community:

*I suppose one way to approach this is by intervention in smaller units like neighbourhoods as opposed to cities or communities or something like that because you’re able to be more focused on what you do (Participant 5).*

## Discussion

Advancement in co-creation research can support the practical and systematic development of more sustained healthy food retail initiatives. This study describes academics’ experiences and perceptions of factors relevant to healthy food retail co-creation research. A social constructionist approach informed this study, showing how understandings of co-creation research in healthy food retail rely on the participants’ worldview and context. This approach implies that actively understanding and considering the social construction of environments is an essential element of future work. This aligns with the ethos of co-creation, which acknowledges individual world views and social construction of meaning towards a shared consensus on the drivers of a problem and active collaboration in developing and implementing solutions.

While co-creation theory clearly articulates a vision of collaborations and interactions, these principles may require more work to identify, operationalise and describe in practice. Our results showed that building collaborative relationships leads to transparent and open communication, well-articulated shared vision, mechanisms that support collaboration, valuing retailers’ knowledge, reciprocal investment (e.g., time, skills, knowledge) and flexibility. The concept of trust was a fundamental pillar of co-creation across relationship building, communication, prioritising and enabling implementation. These practices align with empirical and theoretical literature on collaborations and co-creation, notably health administration. For example, Greenhalgh et al. [29], and Rycroft-Malone, et al. [55], state the importance of empowerment, trust, flexibility and reciprocity for successful co-creation efforts. These attributes do not emerge from a vacuum; they rely on the dynamic nature and complex interdependency of the collaboration and the research process [29, 55], and are commonly left out or abandoned in practice [29].

The identification and alignment of motivations enable the optimisation of stakeholders’ priorities and the design of initiatives that co-create value [56]. Our results provide valuable insight into different retailers’ motivations to participate in co-creation processes (e.g., serve the community, good image, media attention), which can enhance initiatives and align different ways of thinking. Increasing motivations to be involved in the co-creation processes can enhance the process itself, specifically if there is an understanding or identification of a specific type of incentives for participation that can be directed to certain groups [57, 56].

It has been theorised that co-creation may provide a means to understand and optimise public health initiatives as it is a participatory, collaborative, context-sensitive and knowledge-based practice [22, 29]. In the co-creation of health-enabling initiatives in food retail outlets, stakeholders collaborate with different kinds of knowledge, resources and competencies to solve shared problems [20, 58]. Our findings showed that the definition of stakeholder roles can vary depending on expected outcomes, settings and project objectives. Yet, there is a need to determine how health-enabling initiatives can be best created, scaled up or sustained over the long term without the time and capital provided by researchers. One option was increasing the capacity of various actors within food systems.

Evidence shows that asset-based approaches and capacity-building influence co-creation, and that adopting a co-creation approach can improve the system and human capacity [22, 59, 60]. Our results further suggest that capacity-building can be a strategy to sustain initiatives over time. Including capacity building in an initiative (e.g., manager and employee training; structural aspects including new equipment) can enhance social innovation processes and value creation at an individual, organisational and broader societal level [60], which can sustain change.

### Strengths and Limitations

This study adopted a rigorous qualitative approach to interview academics about healthy food retail co-creation research. The development of a codebook guided consistent analysis. Data saturation was reached with the present sample. The various studies and views of academics provided learnings related to the co-creation of health-enabling initiatives in food retail outlets that are relevant for future co-creation theory development and application. One limitation is that participants are leading authors on published research, comprising nine co-creation studies for healthy food retail initiatives with Indigenous peoples or low-income communities in Western countries. The findings may not be generalisable to the 20 studies identified in the review or the broader co-creation effort. However, participants offer experiences that can inform co-creation research in food retail outlets. The purposive sample is naturally limited to published researchers with available contact details and may have introduced further bias (e.g., availability, desirability to participate, or recall bias).

### Future research

Further work should explore the co-creation of health food retail among non-academic participants (e.g., consumers, retailers, and health promotion representatives). Research in this group could enable a comparison of views and perspectives on co-creation research, as well as the identification of how relationships could influence the sustainability of an initiative over time. Additionally, purposively testing a co-creation process could contribute to an innovative and collaborative research design and identify specific needs and evaluation methods of co-creation research in food retail environments.

## Conclusion

Findings from the present study suggest that advancement in co-creation research has the potential to support the development of health-enabling food retail initiatives that are sustained over time and consider the dynamic interaction and relationship between diverse stakeholders (e.g., retailers, consumers, governments) and the context (e.g., business models, policies). The adoption of co-creation varied among participants and provided valuable insights that can help develop healthy food retail co-creation research. Practices such as collaborative relationships based on trusting and respectful relationships, continual interactions and reciprocal acknowledgement between stakeholders are essential in the co-creation process. These practices should be considered in developing and testing a model that helps to systematically co-create healthy food retail initiatives that ensure that all parties meet their needs while also delivering research outcomes.

## Electronic supplementary material

Below is the link to the electronic supplementary material.

Supplementary Material 1

## Data Availability

The interview guide and codebook were provided as additional material. The interview transcripts analysed during the current study are available from the corresponding author on reasonable request and corresponding ethics clearance.
