# Supplementary material for: Co-creation of health-enabling initiatives in food retail: academic perspectives
Source: BMC Public Health. 2023 May 25;23:953. doi: 10.1186/s12889-023-15771-z (PMC10214559; doi:10.1186/s12889-023-15771-z)
Supplement: Supplementary file 1 — Supplementary Material 1 [file 12889_2023_15771_MOESM1_ESM.pdf]

Title

Co-creation of health-enabling initiatives in food retail: academic perspectives

Authors

Carmen Vargas*, Julie Brimblecombe, Steven Allender, Jillian Whelan

*** Corresponding author**

Carmen Vargas

Locked Bag 20000, Geelong, VIC 3220

+61 3 52278039

carmen.vargas@deakin.edu.au

Supplementary File

Table S1. COREQ Checklist 3

Table S2. Interview guide 6

Table S3. Codebook 8

Table S1. COREQ Checklist

A checklist of items that should be included in reports of qualitative research. You must report the page number in your manuscript where you consider each of the items listed in this checklist. If you have not included this information, either revise your manuscript accordingly before submitting or note N/A.

No.	Item	Questions/Description	Reported on: Section
**Domain 1: Research team and reflexivity**			
Personal Characteristics			
1	Interviewer/facilitator	Which author/s conducted the interview or focus group?	Researcher Team and Reflexivity
2	Credentials	What were the researcher's credentials? E.g. PhD, MD	Researcher Team and Reflexivity
3	Occupation	What was their occupation at the time of the study?	Researcher Team and Reflexivity
4	Gender	Was the researcher male or female?	Researcher Team and Reflexivity
5	Experience and training	What experience or training did the researcher have?	Researcher Team and Reflexivity
Relationship with participants			
6	Relationship established	Was a relationship established prior to study commencement?	NA
7	Participant knowledge of the interviewer	What did the participants know about the researcher? e.g. personal goals, reasons for doing the research	Theoretical framework
8	Interviewer characteristics	What characteristics were reported about the interviewer/facilitator? e.g. Bias, assumptions, reasons and interests in the research topic	NA
**Domain 2: study design**			
Theoretical framework			
9	Methodological orientation and Theory	What methodological orientation was stated to underpin the study? e.g. grounded theory, discourse analysis, ethnography, phenomenology, content analysis	Theoretical framework
Participant selection			

No.	Item	Questions/Description	Reported on: Section
10	Sampling	How were participants selected? e.g. purposive, convenience, consecutive, snowball	Participants and recruitment
11	Method of approach	How were participants approached? e.g. face-to-face, telephone, mail, email	Participants and recruitment
12	Sample size	How many participants were in the study?	Results
13	Non-participation	How many people refused to participate or dropped out? Reasons?	Results
Setting			
14	Setting of data collection	Where was the data collected? e.g. home, clinic, workplace	Data collection
15	Presence of non-participants	Was anyone else present besides the participants and researchers?	NA
16	Description of sample	What are the important characteristics of the sample? e.g. demographic data, date	Participants and recruitment
Data Collection			
17	Interview guide	Were questions, prompts, guides provided by the authors? Was it pilot tested?	Data collection
18	Repeat interviews	Were repeat interviews carried out? If yes, how many?	NA
19	Audio/visual recording	Did the research use audio or visual recording to collect the data?	Data collection
20	Field notes	Were field notes made during and/or after the interview or focus group?	Data collection
21	Duration	What was the duration of the interviews or focus group?	Results
22	Data saturation	Was data saturation discussed?	NA
23	Transcripts returned	Were transcripts returned to participants for comment and/or correction?	Data collection
**Domain 3: analysis and findings**			
Data analysis			
24	Number of data coders	How many data coders coded the data?	Data analysis
25	Description of the coding tree	Did authors provide a description of the coding tree?	Supplement Table S3
26	Derivation of themes	Were themes identified in advance or derived from the data?	Data analysis
27	Software	What software, if applicable, was used to manage the data?	Data analysis
28	Participant checking	Did participants provide feedback on the findings?	NA

Reporting			
29	Quotations presented	Were participant quotations presented to illustrate the themes / findings? Was each quotation identified? e.g. participant number	Results
30	Data and findings consistent	Was there consistency between the data presented and the findings?	Results
31	Clarity of major themes	Were major themes clearly presented in the findings?	Supplement Table S3
32	Clarity of minor themes	Is there a description of diverse cases or discussion of minor themes?	Supplement Table S3

*Reported lines relate to the main manuscript document

Developed from: Tong A, Sainsbury P, Craig J. Consolidated criteria for reporting qualitative research (COREQ): a 32-item checklist for interviews and focus groups. International Journal for Quality in Health Care. 2007. 19 (6): 349 – 357

Table S2. Interview guide

<table>
<tr><td colspan="3">Opening/Rapport</td></tr>
<tr><th>Topic</th><th colspan="2">Content</th></tr>
<tr><td>Self-introduction</td><td colspan="2">Name, background</td></tr>
<tr><td>Plain language explanation</td><td colspan="2">Objective of the study and aim of the interview</td></tr>
<tr><td>Record consent for participation</td><td colspan="2">Verbal consent from the participant to be recorded and reiteration of the use of information in a non-identifiable form</td></tr>
<tr><td>Participant introduction</td><td colspan="2">Understanding the participant’s positioning regarding the research topic and experience in food environments research</td></tr>
<tr><td colspan="3">Main interview</td></tr>
<tr><th>Topic</th><th>Rationale</th><th>Type of questions</th></tr>
<tr><td>Healthy food retail outlets' feasibility</td><td>Explore ideas and beliefs on how a healthy food environment looks like</td><td>What do you think a healthy food retail outlet looks like?
How is this idea different or similar to the actual food retail outlet?</td></tr>
<tr><td>Actors involved</td><td>Discussion on the diversity of stakeholders that should be involved in the design of healthy food retail outlet</td><td>If we are to set up a group to work on designing a healthy food retail outlet, who do you believe should be part of this group?</td></tr>
<tr><td>Level of participation</td><td>Discussion of the roles and responsibilities that diverse stakeholders should have</td><td>When do you believe these actors should participate?
Do you think that all actors should collaborate equally?</td></tr>
<tr><td>Interest</td><td>Identify the starting point to initiate the co-creation of food retail outlets.</td><td>Who is more interested in having a healthy food retail outlet?</td></tr>
<tr><td>Motivations</td><td>Explore the drivers that diverse stakeholders may have to co-create healthy food retail outlets</td><td>In your opinion, what could motivate diverse actors to collaborate with others to co-create healthy food retail outlets?</td></tr>
<tr><td>Enablers</td><td>Explore any general or specific enablers for collaboration</td><td>What do you believe would make easier collaboration between diverse stakeholders?</td></tr>
<tr><td>Barriers</td><td>Explore any general or specific barriers/concerns for collaboration</td><td>In your opinion, what type of things would prevent an active</td></tr>
</table>

		collaboration between diverse stakeholders?
Practice	Explore ideas for future practice/planning	In your opinion, what are the next steps required to improve co-creation?

**Closing**	
**Topic**	**Rationale**
Summary of the interview	Summary of main points and final question to add the missing information
Commitment for transcript	A verbal commitment from the interviewer with a timeframe for returning the transcript

Table S3. Codebook

Major Theme	Definition	Minor theme	Definition
1. Stakeholders	Person, group, or organization involved at any point of the initiative.	Roles of collaborators	Comments and considerations related to the level of collaboration of stakeholders (e.g., specific roles)
		Type of stakeholders	Type of stakeholders that should be at the table in the co-creation of initiatives
		Process of engagement	Examples of actions that help the process of engagement in the initial stages of the process,
2. Supporting networks	People/groups that can help achieve the aim. These people can aid the initiative's implementation or help to give continuity to work.[1]	Sustain implementation	People cause or groups are set as structures to sustain initiatives related to food environments.
		Support/advocacy groups	People causes or groups that can help to implement or give buy-in to the initiative
3. Governance	All the processes of interactions (e.g., laws, norms, power or language) of an organized society over a social system through which government and/or non-government actors interact to design and implement policies within a given set of formal and informal sets of rules.[2]	Government policy	Comments related to prescribing or suggested government policies and comments or suggestions about using government policies.
		Organizational policy	Comments related to specific rules or policies within the store, regardless of their relation to improving the healthiness of the store.

Major Theme	Definition	Minor theme	Definition
4. Challenge identification	Determination of the elements for a healthier food retail outlet.	Goal	The main goal is to improve the healthiness of the food retail outlet.
		Healthy retail	Participant's vision of a healthy food retail
5. Strategy/Plan	The specific actions or functions a team takes to support evidence-based practice or program.	Prioritization	Examples and comments related to the elements considered in choosing a specific strategy
		Co-design	Mentions related to experiences with co-design/co-relation throughout the study.
6. Motivations	Individuals desire to achieve a goal, and it is the driving force that makes individuals to action.[3]	Setting-related	Specific considerations of the setting, e.g., political, environmental, population type, etc.)
		Researchers	Comments of participant's drivers to conduct this type of research (e.g., social justice, interest in the method, etc)
7. Communication	Process by which information is developed and/or exchanged between individuals or computers.[1]	Engage	Type of communication-related to initial engagement with the stakeholders
		Exchange	Type of collaboration that can help to build trust between stakeholders
		Inform/dissemination	Type of communication used to inform stakeholders or disseminate the initiative (e.g., consideration to share information with other groups)
8. Evaluation	Any information related to the evaluation of the initiative results of any implementation effort defined in relation to		

Major Theme	Definition	Minor theme	Definition
	improvements in infrastructure, capacity, and impact on the population served by the evidence-based program or practice		
9. Implementation	A specified set of activities designed to put into use a practice or program of known dimensions.[4]	Barriers	Experiences and comments on collaboration/implementation barriers
		Enablers	Experiences and comments of collaboration/implementation enablers
10. Quality improvement	A formal approach to the analysis of practice performance and efforts to improve performance	Evolution of research approaches over time	Participant observations on the way research on food retail environments has evolved.
		Future directions for this research (big picture)	Recommendations for future research at a significant level
		Lessons learned (specific tips and advice)	Comments and recommendations for future practice related to the participant's experiences.
		Sustainability	Information related to the intended benefits of the initiative over an extended period after the implementation is finalised.[5]

References:

1. Manojlovich M, Squires JE, Davies B, Graham ID. Hiding in plain sight: Communication theory in implementation science. Implement Sci. 2015;10(1):58-. 10.1186/s13012-015-0244-y

2. Rhodes RAW. Understanding governance: Ten years on. Organ Stud. 2007;28(8):1243-64. 10.1177/0170840607076586

3. Palma FC, Trimi S, Hong S-G. Motivation triggers for customer participation in value co-creation. Serv Bus. 2019;13(3):557-80. 10.1007/s11628-018-00395-w

4. National Implementation Research Network. Glossary of terms - implementation science [Electronic source]. 2019 [Available from: https://nirn.fpg.unc.edu/glossary-terms-implementation-science.

5. Hailemariam M, Bustos T, Montgomery B, Barajas R, Evans LB, Drahota A. Evidence-based intervention sustainability strategies: A systematic review. Implementation Science. 2019;14(1):57. 10.1186/s13012-019-0910-6
